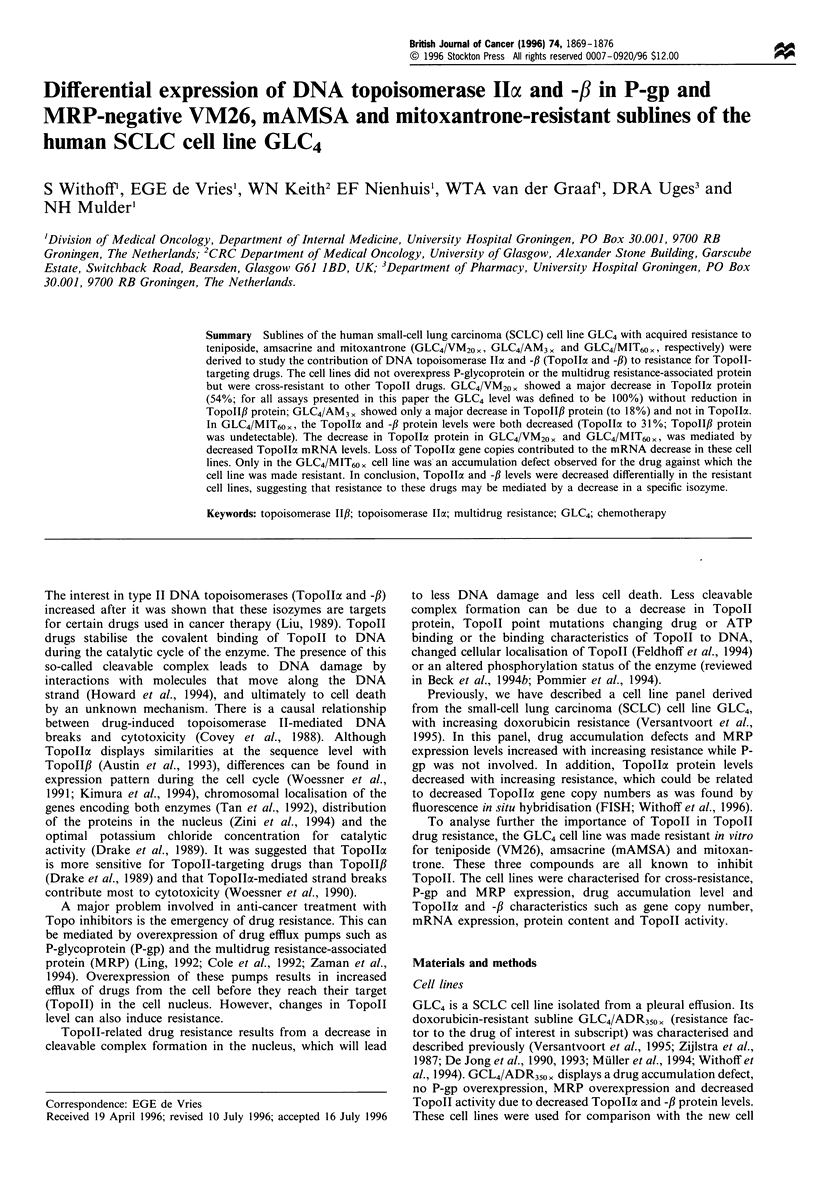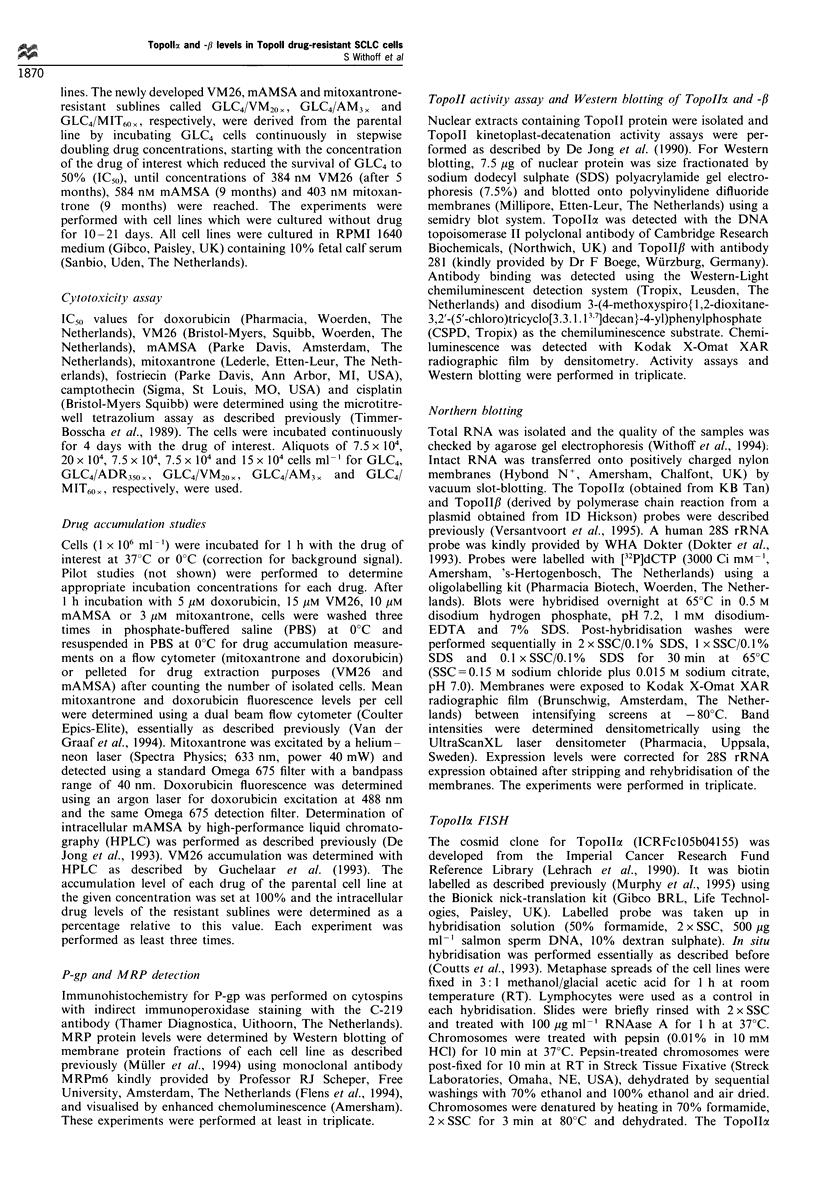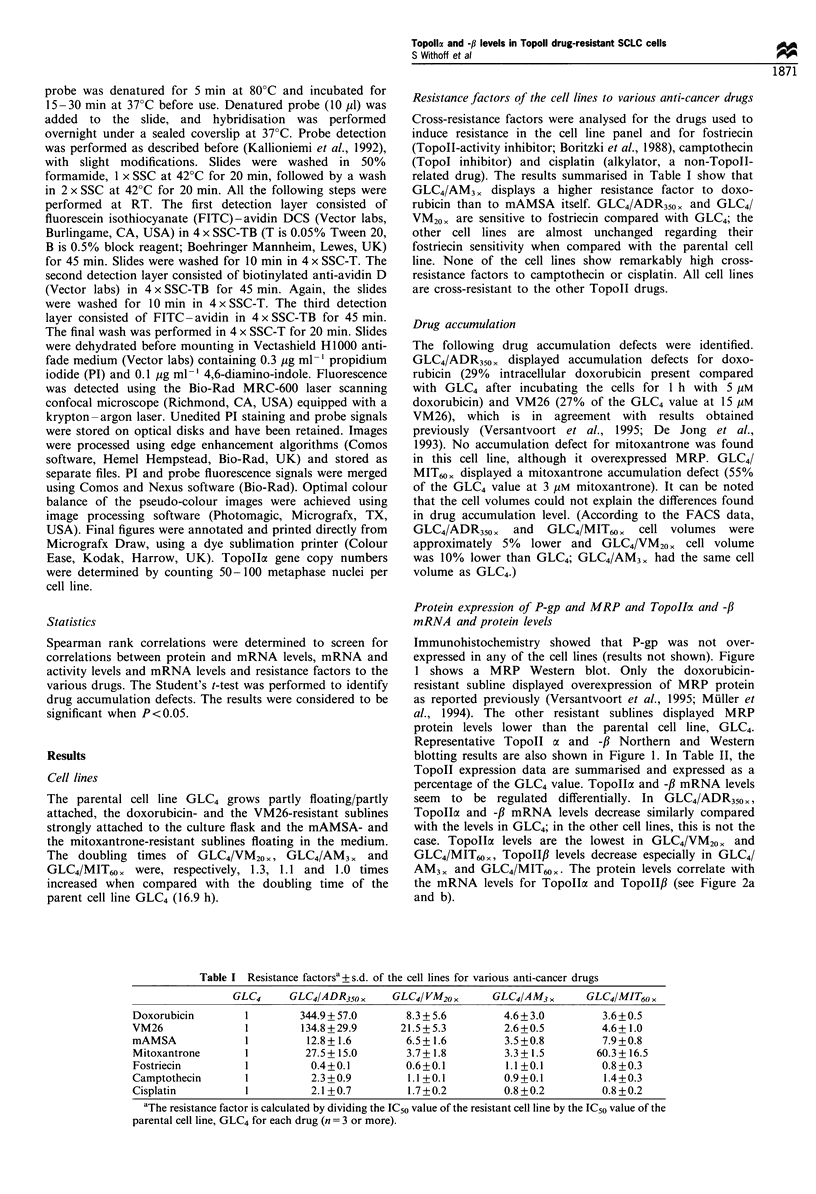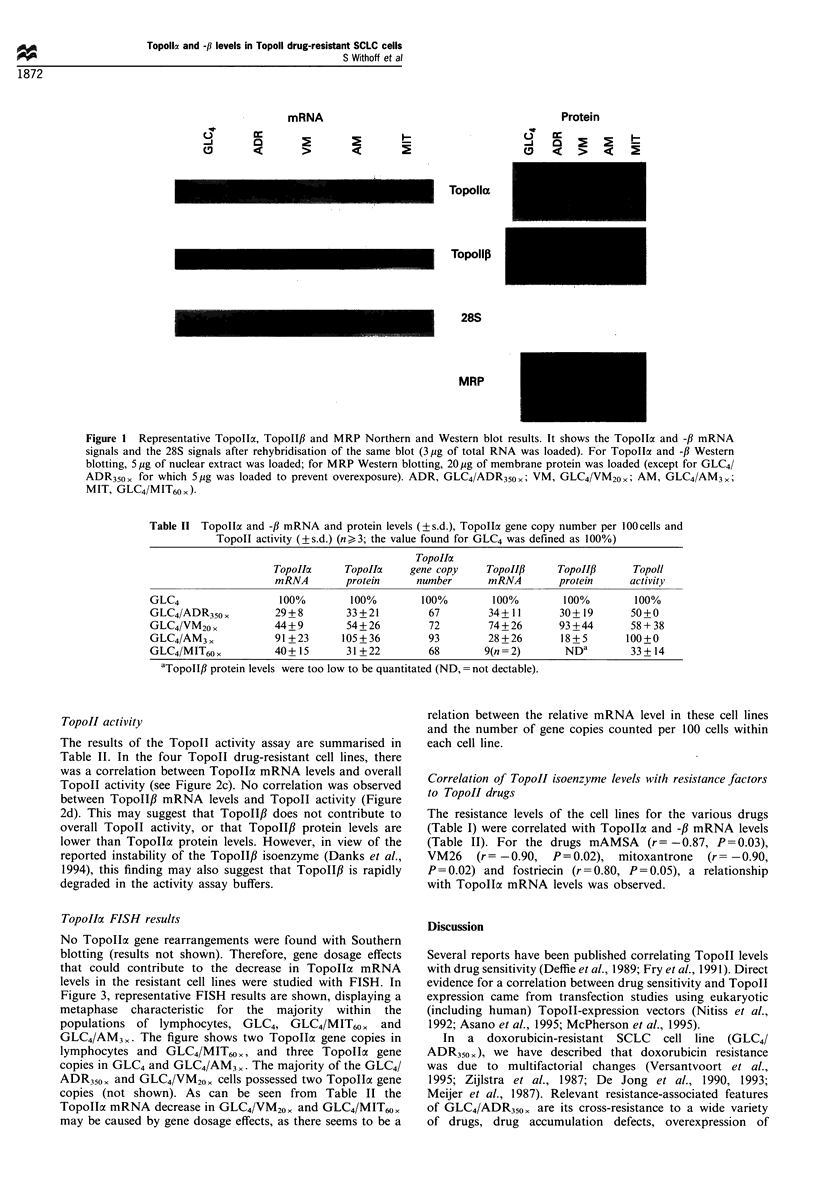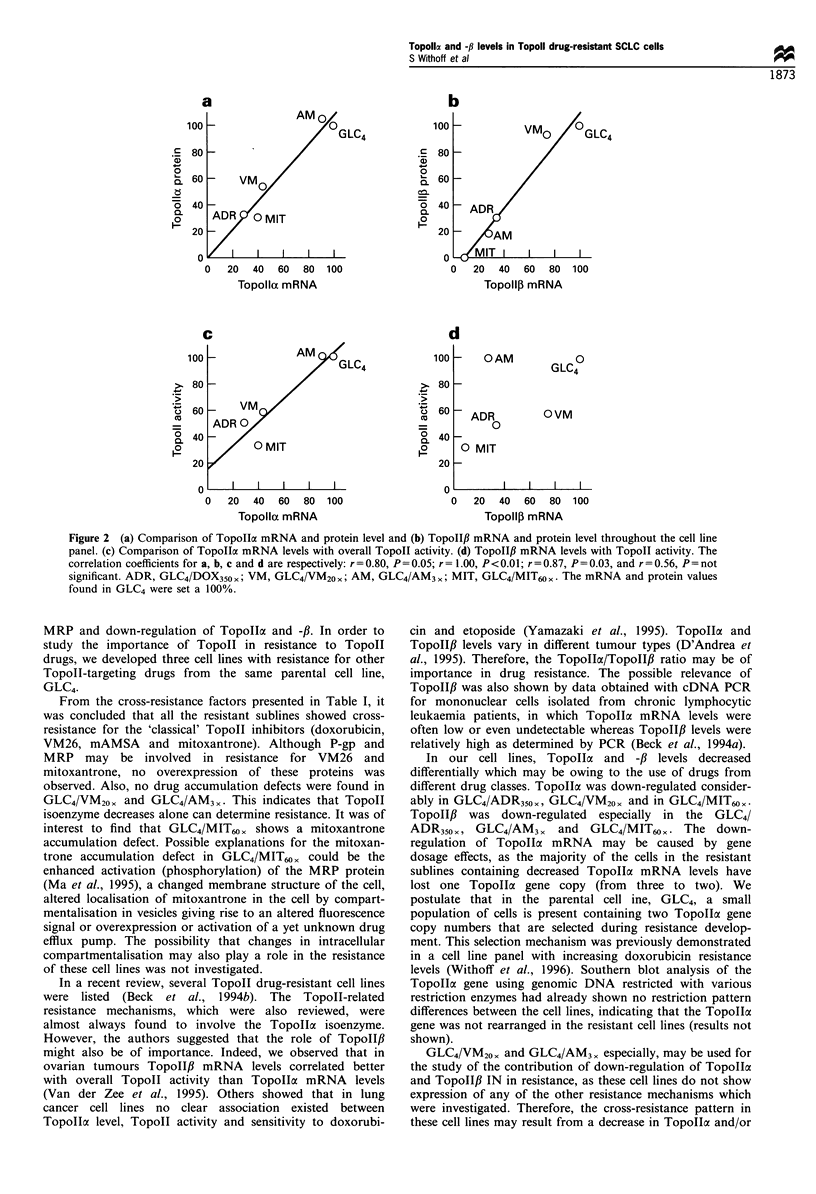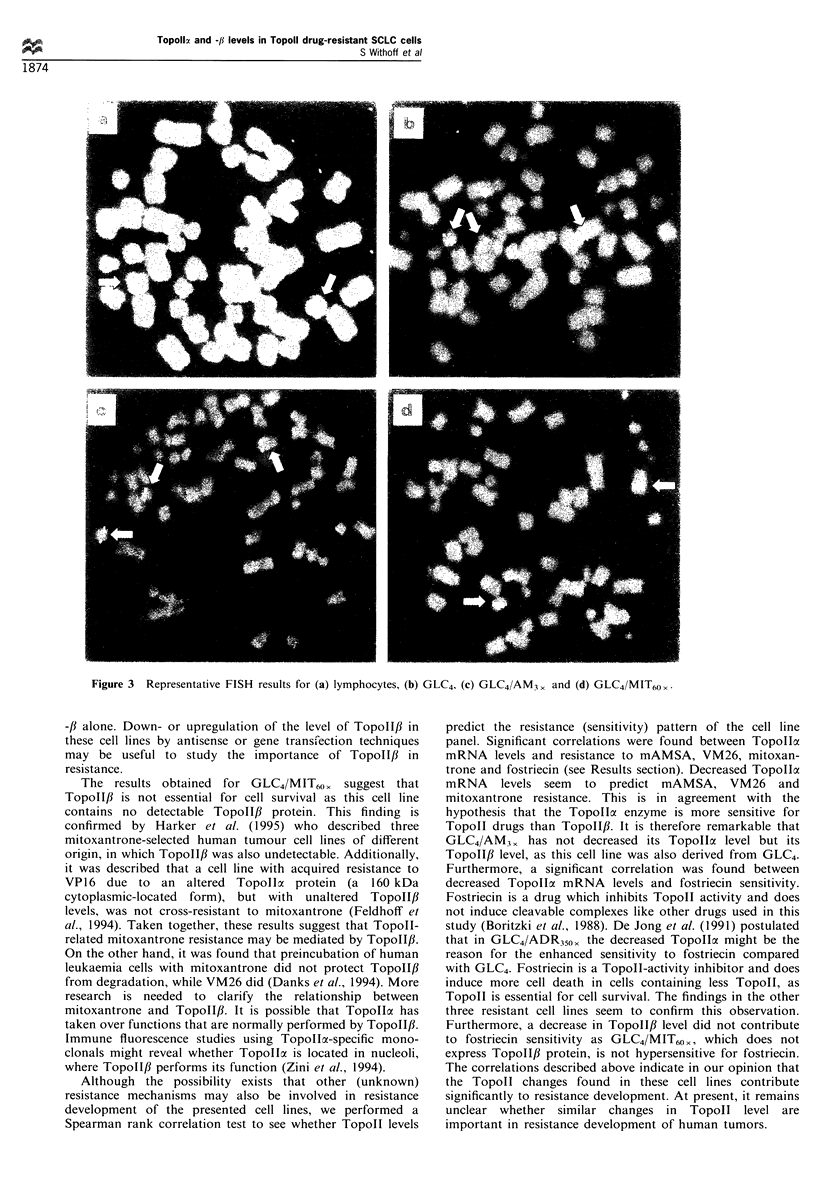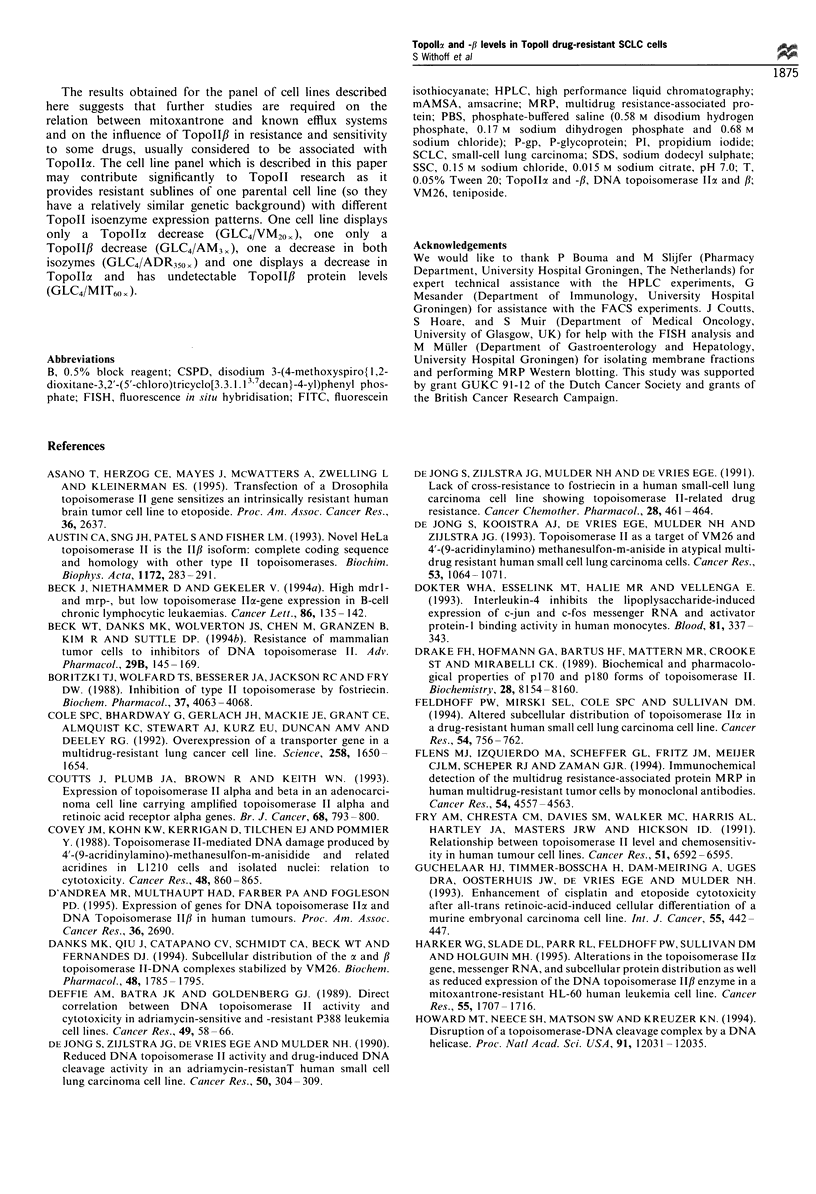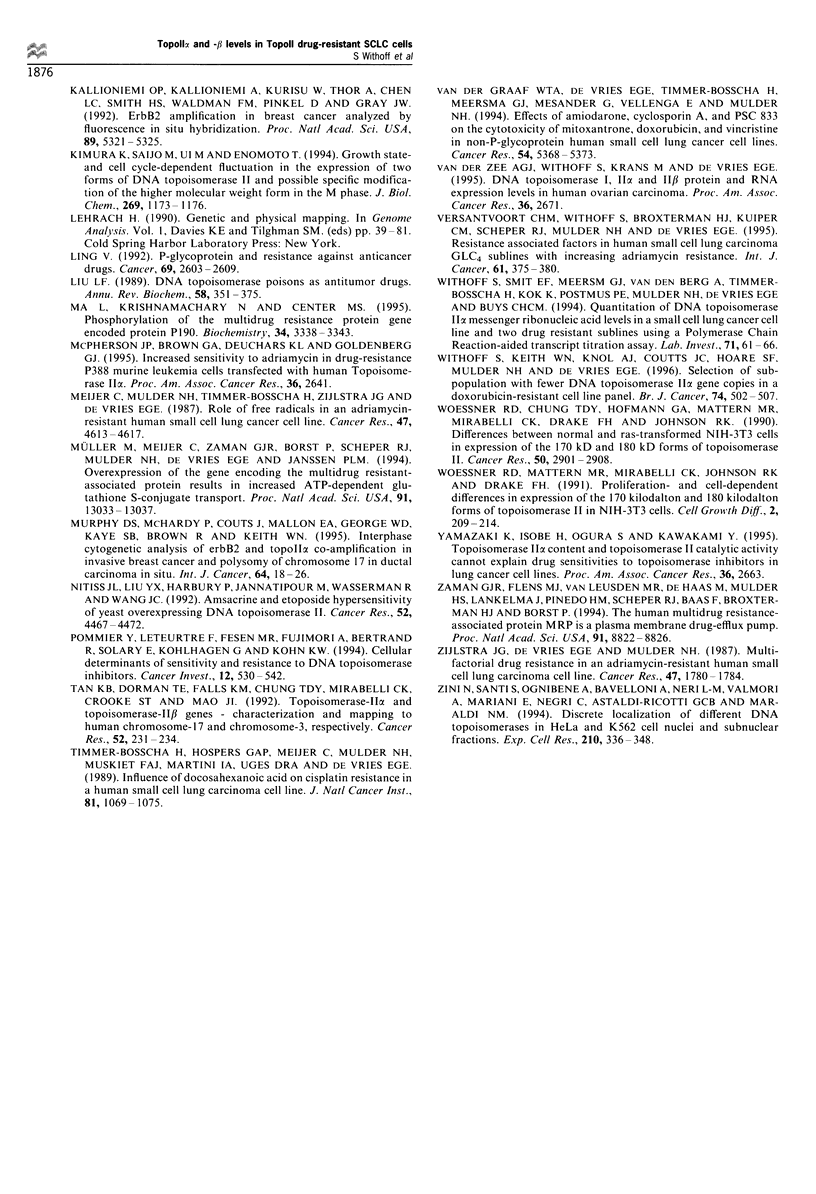# Differential expression of DNA topoisomerase II alpha and -beta in P-gp and MRP-negative VM26, mAMSA and mitoxantrone-resistant sublines of the human SCLC cell line GLC4.

**DOI:** 10.1038/bjc.1996.647

**Published:** 1996-12

**Authors:** S. Withoff, E. G. de Vries, W. N. Keith, E. F. Nienhuis, W. T. van der Graaf, D. R. Uges, N. H. Mulder

**Affiliations:** Department of Internal Medicine, University Hospital Groningen, The Netherlands.

## Abstract

**Images:**


British Journal of Cancer (1996) 74, 1869-1876
© 1996 Stockton Press All rights reserved 0007-0920/96 $12.00

### Differential expression of DNA topoisomerase IIα and -β in P-gp and MRP-negative VM26, mAMSA and mitoxantrone-resistant sublines of the human SCLC cell line $GLC_4$

S Withoff[1], EGE de Vries[1], WN Keith[2] EF Nienhuis[1], WTA van der Graaf[1], DRA Uges[3] and NH Mulder[1]

[1]*Division of Medical Oncology, Department of Internal Medicine, University Hospital Groningen, PO Box 30.001, 9700 RB Groningen, The Netherlands;* [2]*CRC Department of Medical Oncology, University of Glasgow, Alexander Stone Building, Garscube Estate, Switchback Road, Bearsden, Glasgow G61 1BD, UK;* [3]*Department of Pharmacy, University Hospital Groningen, PO Box 30.001, 9700 RB Groningen, The Netherlands.*

**Summary** Sublines of the human small-cell lung carcinoma (SCLC) cell line $GLC_4$ with acquired resistance to teniposide, amsacrine and mitoxantrone ($GLC_4/VM_{20\times}$, $GLC_4/AM_{3\times}$ and $GLC_4/MIT_{60\times}$, respectively) were derived to study the contribution of DNA topoisomerase IIα and -β (TopoIIα and -β) to resistance for TopoII-targeting drugs. The cell lines did not overexpress P-glycoprotein or the multidrug resistance-associated protein but were cross-resistant to other TopoII drugs. $GLC_4/VM_{20\times}$ showed a major decrease in TopoIIα protein (54%; for all assays presented in this paper the $GLC_4$ level was defined to be 100%) without reduction in TopoIIβ protein; $GLC_4/AM_{3\times}$ showed only a major decrease in TopoIIβ protein (to 18%) and not in TopoIIα. In $GLC_4/MIT_{60\times}$, the TopoIIα and -β protein levels were both decreased (TopoIIα to 31%; TopoIIβ protein was undetectable). The decrease in TopoIIα protein in $GLC_4/VM_{20\times}$ and $GLC_4/MIT_{60\times}$, was mediated by decreased TopoIIα mRNA levels. Loss of TopoIIα gene copies contributed to the mRNA decrease in these cell lines. Only in the $GLC_4/MIT_{60\times}$ cell line was an accumulation defect observed for the drug against which the cell line was made resistant. In conclusion, TopoIIα and -β levels were decreased differentially in the resistant cell lines, suggesting that resistance to these drugs may be mediated by a decrease in a specific isozyme.

**Keywords:** topoisomerase IIβ; topoisomerase IIα; multidrug resistance; $GLC_4$; chemotherapy

The interest in type II DNA topoisomerases (TopoIIα and -β) increased after it was shown that these isozymes are targets for certain drugs used in cancer therapy (Liu, 1989). TopoII drugs stabilise the covalent binding of TopoII to DNA during the catalytic cycle of the enzyme. The presence of this so-called cleavable complex leads to DNA damage by interactions with molecules that move along the DNA strand (Howard *et al.*, 1994), and ultimately to cell death by an unknown mechanism. There is a causal relationship between drug-induced topoisomerase II-mediated DNA breaks and cytotoxicity (Covey *et al.*, 1988). Although TopoIIα displays similarities at the sequence level with TopoIIβ (Austin *et al.*, 1993), differences can be found in expression pattern during the cell cycle (Woessner *et al.*, 1991; Kimura *et al.*, 1994), chromosomal localisation of the genes encoding both enzymes (Tan *et al.*, 1992), distribution of the proteins in the nucleus (Zini *et al.*, 1994) and the optimal potassium chloride concentration for catalytic activity (Drake *et al.*, 1989). It was suggested that TopoIIα is more sensitive for TopoII-targeting drugs than TopoIIβ (Drake *et al.*, 1989) and that TopoIIα-mediated strand breaks contribute most to cytotoxicity (Woessner *et al.*, 1990).

A major problem involved in anti-cancer treatment with Topo inhibitors is the emergency of drug resistance. This can be mediated by overexpression of drug efflux pumps such as P-glycoprotein (P-gp) and the multidrug resistance-associated protein (MRP) (Ling, 1992; Cole *et al.*, 1992; Zaman *et al.*, 1994). Overexpression of these pumps results in increased efflux of drugs from the cell before they reach their target (TopoII) in the cell nucleus. However, changes in TopoII level can also induce resistance.

TopoII-related drug resistance results from a decrease in cleavable complex formation in the nucleus, which will lead to less DNA damage and less cell death. Less cleavable complex formation can be due to a decrease in TopoII protein, TopoII point mutations changing drug or ATP binding or the binding characteristics of TopoII to DNA, changed cellular localisation of TopoII (Feldhoff *et al.*, 1994) or an altered phosphorylation status of the enzyme (reviewed in Beck *et al.*, 1994*b*; Pommier *et al.*, 1994).

Previously, we have described a cell line panel derived from the small-cell lung carcinoma (SCLC) cell line $GLC_4$, with increasing doxorubicin resistance (Versantvoort *et al.*, 1995). In this panel, drug accumulation defects and MRP expression levels increased with increasing resistance while P-gp was not involved. In addition, TopoIIα protein levels decreased with increasing resistance, which could be related to decreased TopoIIα gene copy numbers as was found by fluorescence *in situ* hybridisation (FISH; Withoff *et al.*, 1996).

To analyse further the importance of TopoII in TopoII drug resistance, the $GLC_4$ cell line was made resistant *in vitro* for teniposide (VM26), amsacrine (mAMSA) and mitoxantrone. These three compounds are all known to inhibit TopoII. The cell lines were characterised for cross-resistance, P-gp and MRP expression, drug accumulation level and TopoIIα and -β characteristics such as gene copy number, mRNA expression, protein content and TopoII activity.

### Materials and methods

### *Cell lines*

$GLC_4$ is a SCLC cell line isolated from a pleural effusion. Its doxorubicin-resistant subline $GLC_4/ADR_{350\times}$ (resistance factor to the drug of interest in subscript) was characterised and described previously (Versantvoort *et al.*, 1995; Zijlstra *et al.*, 1987; De Jong *et al.*, 1990, 1993; Müller *et al.*, 1994; Withoff *et al.*, 1994). $GCL_4/ADR_{350\times}$ displays a drug accumulation defect, no P-gp overexpression, MRP overexpression and decreased TopoII activity due to decreased TopoIIα and -β protein levels. These cell lines were used for comparison with the new cell

Correspondence: EGE de Vries
Received 19 April 1996; revised 10 July 1996; accepted 16 July 1996

lines. The newly developed VM26, mAMSA and mitoxantrone-resistant sublines called $GLC_4/VM_{20\times}$, $GLC_4/AM_{3\times}$ and $GLC_4/MIT_{60\times}$, respectively, were derived from the parental line by incubating $GLC_4$ cells continuously in stepwise doubling drug concentrations, starting with the concentration of the drug of interest which reduced the survival of $GLC_4$ to 50% ($IC_{50}$), until concentrations of 384 nM VM26 (after 5 months), 584 nM mAMSA (9 months) and 403 nM mitoxantrone (9 months) were reached. The experiments were performed with cell lines which were cultured without drug for 10–21 days. All cell lines were cultured in RPMI 1640 medium (Gibco, Paisley, UK) containing 10% fetal calf serum (Sanbio, Uden, The Netherlands).

*Cytotoxicity assay*

$IC_{50}$ values for doxorubicin (Pharmacia, Woerden, The Netherlands), VM26 (Bristol-Myers, Squibb, Woerden, The Netherlands), mAMSA (Parke Davis, Amsterdam, The Netherlands), mitoxantrone (Lederle, Etten-Leur, The Netherlands), fostriecin (Parke Davis, Ann Arbor, MI, USA), camptothecin (Sigma, St Louis, MO, USA) and cisplatin (Bristol-Myers Squibb) were determined using the microtitre-well tetrazolium assay as described previously (Timmer-Bosscha *et al.*, 1989). The cells were incubated continuously for 4 days with the drug of interest. Aliquots of $7.5 \times 10^4$, $20 \times 10^4$, $7.5 \times 10^4$, $7.5 \times 10^4$ and $15 \times 10^4$ cells $ml^{-1}$ for $GLC_4$, $GLC_4/ADR_{350\times}$, $GLC_4/VM_{20\times}$, $GLC_4/AM_{3\times}$ and $GLC_4/MIT_{60\times}$, respectively, were used.

*Drug accumulation studies*

Cells ($1 \times 10^6$ $ml^{-1}$) were incubated for 1 h with the drug of interest at 37°C or 0°C (correction for background signal). Pilot studies (not shown) were performed to determine appropriate incubation concentrations for each drug. After 1 h incubation with 5 μM doxorubicin, 15 μM VM26, 10 μM mAMSA or 3 μM mitoxantrone, cells were washed three times in phosphate-buffered saline (PBS) at 0°C and resuspended in PBS at 0°C for drug accumulation measurements on a flow cytometer (mitoxantrone and doxorubicin) or pelleted for drug extraction purposes (VM26 and mAMSA) after counting the number of isolated cells. Mean mitoxantrone and doxorubicin fluorescence levels per cell were determined using a dual beam flow cytometer (Coulter Epics-Elite), essentially as described previously (Van der Graaf *et al.*, 1994). Mitoxantrone was excitated by a helium–neon laser (Spectra Physics; 633 nm, power 40 mW) and detected using a standard Omega 675 filter with a bandpass range of 40 nm. Doxorubicin fluorescence was determined using an argon laser for doxorubicin excitation at 488 nm and the same Omega 675 detection filter. Determination of intracellular mAMSA by high-performance liquid chromatography (HPLC) was performed as described previously (De Jong *et al.*, 1993). VM26 accumulation was determined with HPLC as described by Guchelaar *et al.* (1993). The accumulation level of each drug of the parental cell line at the given concentration was set at 100% and the intracellular drug levels of the resistant sublines were determined as a percentage relative to this value. Each experiment was performed as least three times.

*P-gp and MRP detection*

Immunohistochemistry for P-gp was performed on cytospins with indirect immunoperoxidase staining with the C-219 antibody (Thamer Diagnostica, Uithoorn, The Netherlands). MRP protein levels were determined by Western blotting of membrane protein fractions of each cell line as described previously (Müller *et al.*, 1994) using monoclonal antibody MRPm6 kindly provided by Professor RJ Scheper, Free University, Amsterdam, The Netherlands (Flens *et al.*, 1994), and visualised by enhanced chemoluminescence (Amersham). These experiments were performed at least in triplicate.

*TopoII activity assay and Western blotting of TopoIIα and -β*

Nuclear extracts containing TopoII protein were isolated and TopoII kinetoplast-decatenation activity assays were performed as described by De Jong *et al.* (1990). For Western blotting, 7.5 μg of nuclear protein was size fractionated by sodium dodecyl sulphate (SDS) polyacrylamide gel electrophoresis (7.5%) and blotted onto polyvinylidene difluoride membranes (Millipore, Etten-Leur, The Netherlands) using a semidry blot system. TopoIIα was detected with the DNA topoisomerase II polyclonal antibody of Cambridge Research Biochemicals, (Northwich, UK) and TopoIIβ with antibody 281 (kindly provided by Dr F Boege, Würzburg, Germany). Antibody binding was detected using the Western-Light chemiluminescent detection system (Tropix, Leusden, The Netherlands) and disodium 3-(4-methoxyspiro{1,2-dioxitane-3,2′-(5′-chloro)tricyclo[3.3.1.1$^{3,7}$]decan}-4-yl)phenylphosphate (CSPD, Tropix) as the chemiluminescence substrate. Chemiluminescence was detected with Kodak X-Omat XAR radiographic film by densitometry. Activity assays and Western blotting were performed in triplicate.

*Northern blotting*

Total RNA was isolated and the quality of the samples was checked by agarose gel electrophoresis (Withoff *et al.*, 1994). Intact RNA was transferred onto positively charged nylon membranes (Hybond $N^+$, Amersham, Chalfont, UK) by vacuum slot-blotting. The TopoIIα (obtained from KB Tan) and TopoIIβ (derived by polymerase chain reaction from a plasmid obtained from ID Hickson) probes were described previously (Versantvoort *et al.*, 1995). A human 28S rRNA probe was kindly provided by WHA Dokter (Dokter *et al.*, 1993). Probes were labelled with [$^{32}$P]dCTP (3000 Ci $mM^{-1}$, Amersham, 's-Hertogenbosch, The Netherlands) using a oligolabelling kit (Pharmacia Biotech, Woerden, The Netherlands). Blots were hybridised overnight at 65°C in 0.5 M disodium hydrogen phosphate, pH 7.2, 1 mM disodium-EDTA and 7% SDS. Post-hybridisation washes were performed sequentially in $2 \times$SSC/0.1% SDS, $1 \times$SSC/0.1% SDS and $0.1 \times$SSC/0.1% SDS for 30 min at 65°C (SSC = 0.15 M sodium chloride plus 0.015 M sodium citrate, pH 7.0). Membranes were exposed to Kodak X-Omat XAR radiographic film (Brunschwig, Amsterdam, The Netherlands) between intensifying screens at −80°C. Band intensities were determined densitometrically using the UltraScanXL laser densitometer (Pharmacia, Uppsala, Sweden). Expression levels were corrected for 28S rRNA expression obtained after stripping and rehybridisation of the membranes. The experiments were performed in triplicate.

*TopoIIα FISH*

The cosmid clone for TopoIIα (ICRFc105b04155) was developed from the Imperial Cancer Research Fund Reference Library (Lehrach *et al.*, 1990). It was biotin labelled as described previously (Murphy *et al.*, 1995) using the Bionick nick-translation kit (Gibco BRL, Life Technologies, Paisley, UK). Labelled probe was taken up in hybridisation solution (50% formamide, $2 \times$SSC, 500 μg $ml^{-1}$ salmon sperm DNA, 10% dextran sulphate). *In situ* hybridisation was performed essentially as described before (Coutts *et al.*, 1993). Metaphase spreads of the cell lines were fixed in 3:1 methanol/glacial acetic acid for 1 h at room temperature (RT). Lymphocytes were used as a control in each hybridisation. Slides were briefly rinsed with $2 \times$SSC and treated with 100 μg $ml^{-1}$ RNAase A for 1 h at 37°C. Chromosomes were treated with pepsin (0.01% in 10 mM HCl) for 10 min at 37°C. Pepsin-treated chromosomes were post-fixed for 10 min at RT in Streck Tissue Fixative (Streck Laboratories, Omaha, NE, USA), dehydrated by sequential washings with 70% ethanol and 100% ethanol and air dried. Chromosomes were denatured by heating in 70% formamide, $2 \times$SSC for 3 min at 80°C and dehydrated. The TopoIIα

probe was denatured for 5 min at 80°C and incubated for 15–30 min at 37°C before use. Denatured probe (10 μl) was added to the slide, and hybridisation was performed overnight under a sealed coverslip at 37°C. Probe detection was performed as described before (Kallioniemi *et al*., 1992), with slight modifications. Slides were washed in 50% formamide, 1×SSC at 42°C for 20 min, followed by a wash in 2×SSC at 42°C for 20 min. All the following steps were performed at RT. The first detection layer consisted of fluorescein isothiocyanate (FITC)–avidin DCS (Vector labs, Burlingame, CA, USA) in 4×SSC-TB (T is 0.05% Tween 20, B is 0.5% block reagent; Boehringer Mannheim, Lewes, UK) for 45 min. Slides were washed for 10 min in 4×SSC-T. The second detection layer consisted of biotinylated anti-avidin D (Vector labs) in 4×SSC-TB for 45 min. Again, the slides were washed for 10 min in 4×SSC-T. The third detection layer consisted of FITC–avidin in 4×SSC-TB for 45 min. The final wash was performed in 4×SSC-T for 20 min. Slides were dehydrated before mounting in Vectashield H1000 anti-fade medium (Vector labs) containing 0.3 μg $ml^{-1}$ propidium iodide (PI) and 0.1 μg $ml^{-1}$ 4,6-diamino-indole. Fluorescence was detected using the Bio-Rad MRC-600 laser scanning confocal microscope (Richmond, CA, USA) equipped with a krypton–argon laser. Unedited PI staining and probe signals were stored on optical disks and have been retained. Images were processed using edge enhancement algorithms (Comos software, Hemel Hempstead, Bio-Rad, UK) and stored as separate files. PI and probe fluorescence signals were merged using Comos and Nexus software (Bio-Rad). Optimal colour balance of the pseudo-colour images were achieved using image processing software (Photomagic, Micrografx, TX, USA). Final figures were annotated and printed directly from Micrografx Draw, using a dye sublimation printer (Colour Ease, Kodak, Harrow, UK). TopoIIα gene copy numbers were determined by counting 50–100 metaphase nuclei per cell line.

### *Statistics*

Spearman rank correlations were determined to screen for correlations between protein and mRNA levels, mRNA and activity levels and mRNA levels and resistance factors to the various drugs. The Student's *t*-test was performed to identify drug accumulation defects. The results were considered to be significant when $P<0.05$.

### Results

### *Cell lines*

The parental cell line $GLC_4$ grows partly floating/partly attached, the doxorubicin- and the VM26-resistant sublines strongly attached to the culture flask and the mAMSA- and the mitoxantrone-resistant sublines floating in the medium. The doubling times of $GLC_4/VM_{20\times}$, $GLC_4/AM_{3\times}$ and $GLC_4/MIT_{60\times}$ were, respectively, 1.3, 1.1 and 1.0 times increased when compared with the doubling time of the parent cell line $GLC_4$ (16.9 h).

### *Resistance factors of the cell lines to various anti-cancer drugs*

Cross-resistance factors were analysed for the drugs used to induce resistance in the cell line panel and for fostriecin (TopoII-activity inhibitor; Boritzki *et al*., 1988), camptothecin (TopoI inhibitor) and cisplatin (alkylator, a non-TopoII-related drug). The results summarised in Table I show that $GLC_4/AM_{3\times}$ displays a higher resistance factor to doxorubicin than to mAMSA itself. $GLC_4/ADR_{350\times}$ and $GLC_4/VM_{20\times}$ are sensitive to fostriecin compared with $GLC_4$; the other cell lines are almost unchanged regarding their fostriecin sensitivity when compared with the parental cell line. None of the cell lines show remarkably high cross-resistance factors to camptothecin or cisplatin. All cell lines are cross-resistant to the other TopoII drugs.

### *Drug accumulation*

The following drug accumulation defects were identified. $GLC_4/ADR_{350\times}$ displayed accumulation defects for doxorubicin (29% intracellular doxorubicin present compared with $GLC_4$ after incubating the cells for 1 h with 5 μM doxorubicin) and VM26 (27% of the $GLC_4$ value at 15 μM VM26), which is in agreement with results obtained previously (Versantvoort *et al*., 1995; De Jong *et al*., 1993). No accumulation defect for mitoxantrone was found in this cell line, although it overexpressed MRP. $GLC_4/MIT_{60\times}$ displayed a mitoxantrone accumulation defect (55% of the $GLC_4$ value at 3 μM mitoxantrone). It can be noted that the cell volumes could not explain the differences found in drug accumulation level. (According to the FACS data, $GLC_4/ADR_{350\times}$ and $GLC_4/MIT_{60\times}$ cell volumes were approximately 5% lower and $GLC_4/VM_{20\times}$ cell volume was 10% lower than $GLC_4$; $GLC_4/AM_{3\times}$ had the same cell volume as $GLC_4$.)

### *Protein expression of P-gp and MRP and TopoIIα and -β mRNA and protein levels*

Immunohistochemistry showed that P-gp was not over-expressed in any of the cell lines (results not shown). Figure 1 shows a MRP Western blot. Only the doxorubicin-resistant subline displayed overexpression of MRP protein as reported previously (Versantvoort *et al*., 1995; Müller *et al*., 1994). The other resistant sublines displayed MRP protein levels lower than the parental cell line, $GLC_4$. Representative TopoII α and -β Northern and Western blotting results are also shown in Figure 1. In Table II, the TopoII expression data are summarised and expressed as a percentage of the $GLC_4$ value. TopoIIα and -β mRNA levels seem to be regulated differentially. In $GLC_4/ADR_{350\times}$, TopoIIα and -β mRNA levels decrease similarly compared with the levels in $GLC_4$; in the other cell lines, this is not the case. TopoIIα levels are the lowest in $GLC_4/VM_{20\times}$ and $GLC_4/MIT_{60\times}$, TopoIIβ levels decrease especially in $GLC_4/AM_{3\times}$ and $GLC_4/MIT_{60\times}$. The protein levels correlate with the mRNA levels for TopoIIα and TopoIIβ (see Figure 2a and b).

**Table I** Resistance factors[a] ± s.d. of the cell lines for various anti-cancer drugs

| | $GLC_4$ | $GLC_4/ADR_{350\times}$ | $GLC_4/VM_{20\times}$ | $GLC_4/AM_{3\times}$ | $GLC_4/MIT_{60\times}$ |
|---|---|---|---|---|---|
| Doxorubicin | 1 | 344.9±57.0 | 8.3±5.6 | 4.6±3.0 | 3.6±0.5 |
| VM26 | 1 | 134.8±29.9 | 21.5±5.3 | 2.6±0.5 | 4.6±1.0 |
| mAMSA | 1 | 12.8±1.6 | 6.5±1.6 | 3.5±0.8 | 7.9±0.8 |
| Mitoxantrone | 1 | 27.5±15.0 | 3.7±1.8 | 3.3±1.5 | 60.3±16.5 |
| Fostriecin | 1 | 0.4±0.1 | 0.6±0.1 | 1.1±0.1 | 0.8±0.3 |
| Camptothecin | 1 | 2.3±0.9 | 1.1±0.1 | 0.9±0.1 | 1.4±0.3 |
| Cisplatin | 1 | 2.1±0.7 | 1.7±0.2 | 0.8±0.2 | 0.8±0.2 |

[a]The resistance factor is calculated by dividing the $IC_{50}$ value of the resistant cell line by the $IC_{50}$ value of the parental cell line, $GLC_4$ for each drug ($n=3$ or more).

**Figure 1** Representative TopoIIα, TopoIIβ and MRP Northern and Western blot results. It shows the TopoIIα and -β mRNA signals and the 28S signals after rehybridisation of the same blot (3 μg of total RNA was loaded). For TopoIIα and -β Western blotting, 5 μg of nuclear extract was loaded; for MRP Western blotting, 20 μg of membrane protein was loaded (except for $GLC_4/ADR_{350\times}$ for which 5 μg was loaded to prevent overexposure). ADR, $GLC_4/ADR_{350\times}$; VM, $GLC_4/VM_{20\times}$; AM, $GLC_4/AM_{3\times}$; MIT, $GLC_4/MIT_{60\times}$).

**Table II** TopoIIα and -β mRNA and protein levels (±s.d.), TopoIIα gene copy number per 100 cells and TopoII activity (±s.d.) ($n \geq 3$; the value found for $GLC_4$ was defined as 100%)

| | *TopoIIα mRNA* | *TopoIIα protein* | *TopoIIα gene copy number* | *TopoIIβ mRNA* | *TopoIIβ protein* | *TopoII activity* |
|---|---|---|---|---|---|---|
| $GLC_4$ | 100% | 100% | 100% | 100% | 100% | 100% |
| $GLC_4/ADR_{350\times}$ | 29±8 | 33±21 | 67 | 34±11 | 30±19 | 50±0 |
| $GLC_4/VM_{20\times}$ | 44±9 | 54±26 | 72 | 74±26 | 93±44 | 58+38 |
| $GLC_4/AM_{3\times}$ | 91±23 | 105±36 | 93 | 28±26 | 18±5 | 100±0 |
| $GLC_4/MIT_{60\times}$ | 40±15 | 31±22 | 68 | 9($n$=2) | ND[a] | 33±14 |

[a]TopoIIβ protein levels were too low to be quantitated (ND, = not dectable).

*TopoII activity*

The results of the TopoII activity assay are summarised in Table II. In the four TopoII drug-resistant cell lines, there was a correlation between TopoIIα mRNA levels and overall TopoII activity (see Figure 2c). No correlation was observed between TopoIIβ mRNA levels and TopoII activity (Figure 2d). This may suggest that TopoIIβ does not contribute to overall TopoII activity, or that TopoIIβ protein levels are lower than TopoIIα protein levels. However, in view of the reported instability of the TopoIIβ isoenzyme (Danks *et al.*, 1994), this finding may also suggest that TopoIIβ is rapidly degraded in the activity assay buffers.

*TopoIIα FISH results*

No TopoIIα gene rearrangements were found with Southern blotting (results not shown). Therefore, gene dosage effects that could contribute to the decrease in TopoIIα mRNA levels in the resistant cell lines were studied with FISH. In Figure 3, representative FISH results are shown, displaying a metaphase characteristic for the majority within the populations of lymphocytes, $GLC_4$, $GLC_4/MIT_{60\times}$ and $GLC_4/AM_{3\times}$. The figure shows two TopoIIα gene copies in lymphocytes and $GLC_4/MIT_{60\times}$, and three TopoIIα gene copies in $GLC_4$ and $GLC_4/AM_{3\times}$. The majority of the $GLC_4/ADR_{350\times}$ and $GLC_4/VM_{20\times}$ cells possessed two TopoIIα gene copies (not shown). As can be seen from Table II the TopoIIα mRNA decrease in $GLC_4/VM_{20\times}$ and $GLC_4/MIT_{60\times}$ may be caused by gene dosage effects, as there seems to be a relation between the relative mRNA level in these cell lines and the number of gene copies counted per 100 cells within each cell line.

*Correlation of TopoII isoenzyme levels with resistance factors to TopoII drugs*

The resistance levels of the cell lines for the various drugs (Table I) were correlated with TopoIIα and -β mRNA levels (Table II). For the drugs mAMSA ($r = -0.87$, $P = 0.03$), VM26 ($r = -0.90$, $P = 0.02$), mitoxantrone ($r = -0.90$, $P = 0.02$) and fostriecin ($r = 0.80$, $P = 0.05$), a relationship with TopoIIα mRNA levels was observed.

### Discussion

Several reports have been published correlating TopoII levels with drug sensitivity (Deffie *et al.*, 1989; Fry *et al.*, 1991). Direct evidence for a correlation between drug sensitivity and TopoII expression came from transfection studies using eukaryotic (including human) TopoII-expression vectors (Nitiss *et al.*, 1992; Asano *et al.*, 1995; McPherson *et al.*, 1995).

In a doxorubicin-resistant SCLC cell line ($GLC_4/ADR_{350\times}$), we have described that doxorubicin resistance was due to multifactorial changes (Versantvoort *et al.*, 1995; Zijlstra *et al.*, 1987; De Jong *et al.*, 1990, 1993; Meijer *et al.*, 1987). Relevant resistance-associated features of $GLC_4/ADR_{350\times}$ are its cross-resistance to a wide variety of drugs, drug accumulation defects, overexpression of

**Figure 2** (**a**) Comparison of TopoII$\alpha$ mRNA and protein level and (**b**) TopoII$\beta$ mRNA and protein level throughout the cell line panel. (**c**) Comparison of TopoII$\alpha$ mRNA levels with overall TopoII activity. (**d**) TopoII$\beta$ mRNA levels with TopoII activity. The correlation coefficients for **a**, **b**, **c** and **d** are respectively: $r=0.80$, $P=0.05$; $r=1.00$, $P<0.01$; $r=0.87$, $P=0.03$, and $r=0.56$, $P=$not significant. ADR, $GLC_4/DOX_{350\times}$; VM, $GLC_4/VM_{20\times}$; AM, $GLC_4/AM_{3\times}$; MIT, $GLC_4/MIT_{60\times}$. The mRNA and protein values found in $GLC_4$ were set a 100%.

MRP and down-regulation of TopoII$\alpha$ and -$\beta$. In order to study the importance of TopoII in resistance to TopoII drugs, we developed three cell lines with resistance for other TopoII-targeting drugs from the same parental cell line, $GLC_4$.

From the cross-resistance factors presented in Table I, it was concluded that all the resistant sublines showed cross-resistance for the 'classical' TopoII inhibitors (doxorubicin, VM26, mAMSA and mitoxantrone). Although P-gp and MRP may be involved in resistance for VM26 and mitoxantrone, no overexpression of these proteins was observed. Also, no drug accumulation defects were found in $GLC_4/VM_{20\times}$ and $GLC_4/AM_{3\times}$. This indicates that TopoII isoenzyme decreases alone can determine resistance. It was of interest to find that $GLC_4/MIT_{60\times}$ shows a mitoxantrone accumulation defect. Possible explanations for the mitoxantrone accumulation defect in $GLC_4/MIT_{60\times}$ could be the enhanced activation (phosphorylation) of the MRP protein (Ma *et al.*, 1995), a changed membrane structure of the cell, altered localisation of mitoxantrone in the cell by compartmentalisation in vesicles giving rise to an altered fluorescence signal or overexpression or activation of a yet unknown drug efflux pump. The possibility that changes in intracellular compartmentalisation may also play a role in the resistance of these cell lines was not investigated.

In a recent review, several TopoII drug-resistant cell lines were listed (Beck *et al.*, 1994*b*). The TopoII-related resistance mechanisms, which were also reviewed, were almost always found to involve the TopoII$\alpha$ isoenzyme. However, the authors suggested that the role of TopoII$\beta$ might also be of importance. Indeed, we observed that in ovarian tumours TopoII$\beta$ mRNA levels correlated better with overall TopoII activity than TopoII$\alpha$ mRNA levels (Van der Zee *et al.*, 1995). Others showed that in lung cancer cell lines no clear association existed between TopoII$\alpha$ level, TopoII activity and sensitivity to doxorubicin and etoposide (Yamazaki *et al.*, 1995). TopoII$\alpha$ and TopoII$\beta$ levels vary in different tumour types (D'Andrea *et al.*, 1995). Therefore, the TopoII$\alpha$/TopoII$\beta$ ratio may be of importance in drug resistance. The possible relevance of TopoII$\beta$ was also shown by data obtained with cDNA PCR for mononuclear cells isolated from chronic lymphocytic leukaemia patients, in which TopoII$\alpha$ mRNA levels were often low or even undetectable whereas TopoII$\beta$ levels were relatively high as determined by PCR (Beck *et al.*, 1994*a*).

In our cell lines, TopoII$\alpha$ and -$\beta$ levels decreased differentially which may be owing to the use of drugs from different drug classes. TopoII$\alpha$ was down-regulated considerably in $GLC_4/ADR_{350\times}$, $GLC_4/VM_{20\times}$ and in $GLC_4/MIT_{60\times}$. TopoII$\beta$ was down-regulated especially in the $GLC_4/ADR_{350\times}$, $GLC_4/AM_{3\times}$ and $GLC_4/MIT_{60\times}$. The down-regulation of TopoII$\alpha$ mRNA may be caused by gene dosage effects, as the majority of the cells in the resistant sublines containing decreased TopoII$\alpha$ mRNA levels have lost one TopoII$\alpha$ gene copy (from three to two). We postulate that in the parental cell ine, $GLC_4$, a small population of cells is present containing two TopoII$\alpha$ gene copy numbers that are selected during resistance development. This selection mechanism was previously demonstrated in a cell line panel with increasing doxorubicin resistance levels (Withoff *et al.*, 1996). Southern blot analysis of the TopoII$\alpha$ gene using genomic DNA restricted with various restriction enzymes had already shown no restriction pattern differences between the cell lines, indicating that the TopoII$\alpha$ gene was not rearranged in the resistant cell lines (results not shown).

$GLC_4/VM_{20\times}$ and $GLC_4/AM_{3\times}$ especially, may be used for the study of the contribution of down-regulation of TopoII$\alpha$ and TopoII$\beta$ IN in resistance, as these cell lines do not show expression of any of the other resistance mechanisms which were investigated. Therefore, the cross-resistance pattern in these cell lines may result from a decrease in TopoII$\alpha$ and/or

**Figure 3** Representative FISH results for (**a**) lymphocytes, (**b**) $GLC_4$, (**c**) $GLC_4/AM_{3\times}$ and (**d**) $GLC_4/MIT_{60\times}$.

-β alone. Down- or upregulation of the level of TopoIIβ in these cell lines by antisense or gene transfection techniques may be useful to study the importance of TopoIIβ in resistance.

The results obtained for $GLC_4/MIT_{60\times}$ suggest that TopoIIβ is not essential for cell survival as this cell line contains no detectable TopoIIβ protein. This finding is confirmed by Harker *et al.* (1995) who described three mitoxantrone-selected human tumour cell lines of different origin, in which TopoIIβ was also undetectable. Additionally, it was described that a cell line with acquired resistance to VP16 due to an altered TopoIIα protein (a 160 kDa cytoplasmic-located form), but with unaltered TopoIIβ levels, was not cross-resistant to mitoxantrone (Feldhoff *et al.*, 1994). Taken together, these results suggest that TopoII-related mitoxantrone resistance may be mediated by TopoIIβ. On the other hand, it was found that preincubation of human leukaemia cells with mitoxantrone did not protect TopoIIβ from degradation, while VM26 did (Danks *et al.*, 1994). More research is needed to clarify the relationship between mitoxantrone and TopoIIβ. It is possible that TopoIIα has taken over functions that are normally performed by TopoIIβ. Immune fluorescence studies using TopoIIα-specific monoclonals might reveal whether TopoIIα is located in nucleoli, where TopoIIβ performs its function (Zini *et al.*, 1994).

Although the possibility exists that other (unknown) resistance mechanisms may also be involved in resistance development of the presented cell lines, we performed a Spearman rank correlation test to see whether TopoII levels predict the resistance (sensitivity) pattern of the cell line panel. Significant correlations were found between TopoIIα mRNA levels and resistance to mAMSA, VM26, mitoxantrone and fostriecin (see Results section). Decreased TopoIIα mRNA levels seem to predict mAMSA, VM26 and mitoxantrone resistance. This is in agreement with the hypothesis that the TopoIIα enzyme is more sensitive for TopoII drugs than TopoIIβ. It is therefore remarkable that $GLC_4/AM_{3\times}$ has not decreased its TopoIIα level but its TopoIIβ level, as this cell line was also derived from $GLC_4$. Furthermore, a significant correlation was found between decreased TopoIIα mRNA levels and fostriecin sensitivity. Fostriecin is a drug which inhibits TopoII activity and does not induce cleavable complexes like other drugs used in this study (Boritzki *et al.*, 1988). De Jong *et al.* (1991) postulated that in $GLC_4/ADR_{350\times}$ the decreased TopoIIα might be the reason for the enhanced sensitivity to fostriecin compared with $GLC_4$. Fostriecin is a TopoII-activity inhibitor and does induce more cell death in cells containing less TopoII, as TopoII is essential for cell survival. The findings in the other three resistant cell lines seem to confirm this observation. Furthermore, a decrease in TopoIIβ level did not contribute to fostriecin sensitivity as $GLC_4/MIT_{60\times}$, which does not express TopoIIβ protein, is not hypersensitive for fostriecin. The correlations described above indicate in our opinion that the TopoII changes found in these cell lines contribute significantly to resistance development. At present, it remains unclear whether similar changes in TopoII level are important in resistance development of human tumors.

The results obtained for the panel of cell lines described here suggests that further studies are required on the relation between mitoxantrone and known efflux systems and on the influence of TopoIIβ in resistance and sensitivity to some drugs, usually considered to be associated with TopoIIα. The cell line panel which is described in this paper may contribute significantly to TopoII research as it provides resistant sublines of one parental cell line (so they have a relatively similar genetic background) with different TopoII isoenzyme expression patterns. One cell line displays only a TopoIIα decrease ($GLC_4/VM_{20\times}$), one only a TopoIIβ decrease ($GLC_4/AM_{3\times}$), one a decrease in both isozymes ($GLC_4/ADR_{350\times}$) and one displays a decrease in TopoIIα and has undetectable TopoIIβ protein levels ($GLC_4/MIT_{60\times}$).

### Abbreviations

B, 0.5% block reagent; CSPD, disodium 3-(4-methoxyspiro{1,2-dioxitane-3,2′-(5′-chloro)tricyclo[3.3.1.$1^{3,7}$decan}-4-yl)phenyl phosphate; FISH, fluorescence *in situ* hybridisation; FITC, fluorescein isothiocyanate; HPLC, high performance liquid chromatography; mAMSA, amsacrine; MRP, multidrug resistance-associated protein; PBS, phosphate-buffered saline (0.58 M disodium hydrogen phosphate, 0.17 M sodium dihydrogen phosphate and 0.68 M sodium chloride); P-gp, P-glycoprotein; PI, propidium iodide; SCLC, small-cell lung carcinoma; SDS, sodium dodecyl sulphate; SSC, 0.15 M sodium chloride, 0.015 M sodium citrate, pH 7.0; T, 0.05% Tween 20; TopoIIα and -β, DNA topoisomerase IIα and β; VM26, teniposide.

### Acknowledgements

We would like to thank P Bouma and M Slijfer (Pharmacy Department, University Hospital Groningen, The Netherlands) for expert technical assistance with the HPLC experiments, G Mesander (Department of Immunology, University Hospital Groningen) for assistance with the FACS experiments. J Coutts, S Hoare, and S Muir (Department of Medical Oncology, University of Glasgow, UK) for help with the FISH analysis and M Müller (Department of Gastroenterology and Hepatology, University Hospital Groningen) for isolating membrane fractions and performing MRP Western blotting. This study was supported by grant GUKC 91-12 of the Dutch Cancer Society and grants of the British Cancer Research Campaign.

### References

ASANO T, HERZOG CE, MAYES J, MCWATTERS A, ZWELLING L AND KLEINERMAN ES. (1995). Transfection of a Drosophila topoisomerase II gene sensitizes an intrinsically resistant human brain tumor cell line to etoposide. *Proc. Am. Assoc. Cancer Res.*, **36,** 2637.

AUSTIN CA, SNG JH, PATEL S AND FISHER LM. (1993). Novel HeLa topoisomerase II is the IIβ isoform: complete coding sequence and homology with other type II topoisomerases. *Biochim. Biophys. Acta*, **1172,** 283–291.

BECK J, NIETHAMMER D AND GEKELER V. (1994*a*). High mdr1- and mrp-, but low topoisomerase IIα-gene expression in B-cell chronic lymphocytic leukaemias. *Cancer Lett.*, **86,** 135–142.

BECK WT, DANKS MK, WOLVERTON JS, CHEN M, GRANZEN B, KIM R AND SUTTLE DP. (1994*b*). Resistance of mammalian tumor cells to inhibitors of DNA topoisomerase II. *Adv. Pharmacol.*, **29B,** 145–169.

BORITZKI TJ, WOLFARD TS, BESSERER JA, JACKSON RC AND FRY DW. (1988). Inhibition of type II topoisomerase expression by fostriecin. *Biochem. Pharmacol.*, **37,** 4063–4068.

COLE SPC, BHARDWAY G, GERLACH JH, MACKIE JE, GRANT CE, ALMQUIST KC, STEWART AJ, KURZ EU, DUNCAN AMV AND DEELEY RG. (1992). Overexpression of a transporter gene in a multidrug-resistant lung cancer cell line. *Science*, **258,** 1650–1654.

COUTTS J, PLUMB JA, BROWN R AND KEITH WN. (1993). Expression of topoisomerase II alpha and beta in an adenocarcinoma cell line carrying amplified topoisomerase II alpha and retinoic acid receptor alpha genes. *Br. J. Cancer*, **68,** 793–800.

COVEY JM, KOHN KW, KERRIGAN D, TILCHEN EJ AND POMMIER Y. (1988). Topoisomerase II-mediated DNA damage produced by 4′-(9-acridinylamino)-methanesulfon-m-anisidide and related acridines in L1210 cells and isolated nuclei: relation to cytotoxicity. *Cancer Res.*, **48,** 860–865.

D'ANDREA MR, MULTHAUPT HAD, FARBER PA AND FOGLESON PD. (1995). Expression of genes for DNA topoisomerase IIα and DNA Topoisomerase IIβ in human tumours. *Proc. Am. Assoc. Cancer Res.*, **36,** 2690.

DANKS MK, QIU J, CATAPANO CV, SCHMIDT CA, BECK WT AND FERNANDES DJ. (1994). Subcellular distribution of the α and β topoisomerase II-DNA complexes stabilized by VM26. *Biochem. Pharmacol.*, **48,** 1785–1795.

DEFFIE AM, BATRA JK AND GOLDENBERG GJ. (1989). Direct correlation between DNA topoisomerase II activity and cytotoxicity in adriamycin-sensitive and -resistant P388 leukemia cell lines. *Cancer Res.*, **49,** 58–66.

DE JONG S, ZIJLSTRA JG, DE VRIES EGE AND MULDER NH. (1990). Reduced DNA topoisomerase II activity and drug-induced DNA cleavage activity in an adriamycin-resistanT human small cell lung carcinoma cell line. *Cancer Res.*, **50,** 304–309.

DE JONG S, ZIJLSTRA JG, MULDER NH AND DE VRIES EGE. (1991). Lack of cross-resistance to fostriecin in a human small-cell lung carcinoma cell line showing topoisomerase II-related drug resistance. *Cancer Chemother. Pharmacol.*, **28,** 461–464.

DE JONG S, KOOISTRA AJ, DE VRIES EGE, MULDER NH AND ZIJLSTRA JG. (1993). Topoisomerase II as a target of VM26 and 4′-(9-acridinylamino) methanesulfon-m-aniside in atypical multidrug resistant human small cell lung carcinoma cells. *Cancer Res.*, **53,** 1064–1071.

DOKTER WHA, ESSELINK MT, HALIE MR AND VELLENGA E. (1993). Interleukin-4 inhibits the lipoplysaccharide-induced expression of c-jun and c-fos messenger RNA and activator protein-1 binding activity in human monocytes. *Blood*, **81,** 337–343.

DRAKE FH, HOFMANN GA, BARTUS HF, MATTERN MR, CROOKE ST AND MIRABELLI CK. (1989). Biochemical and pharmacological properties of p170 and p180 forms of topoisomerase II. *Biochemistry*, **28,** 8154–8160.

FELDHOFF PW, MIRSKI SEL, COLE SPC AND SULLIVAN DM. (1994). Altered subcellular distribution of topoisomerase IIα in a drug-resistant human small cell lung carcinoma cell line. *Cancer Res.*, **54,** 756–762.

FLENS MJ, IZQUIERDO MA, SCHEFFER GL, FRITZ JM, MEIJER CJLM, SCHEPER RJ AND ZAMAN GJR. (1994). Immunochemical detection of the multidrug resistance-associated protein MRP in human multidrug-resistant tumor cells by monoclonal antibodies. *Cancer Res.*, **54,** 4557–4563.

FRY AM, CHRESTA CM, DAVIES SM, WALKER MC, HARRIS AL, HARTLEY JA, MASTERS JRW AND HICKSON ID. (1991). Relationship between topoisomerase II level and chemosensitivity in human tumour cell lines. *Cancer Res.*, **51,** 6592–6595.

GUCHELAAR HJ, TIMMER-BOSSCHA H, DAM-MEIRING A, UGES DRA, OOSTERHUIS JW, DE VRIES EGE AND MULDER NH. (1993). Enhancement of cisplatin and etoposide cytotoxicity after all-trans retinoic-acid-induced cellular differentiation of a murine embryonal carcinoma cell line. *Int. J. Cancer*, **55,** 442–447.

HARKER WG, SLADE DL, PARR RL, FELDHOFF PW, SULLIVAN DM AND HOLGUIN MH. (1995). Alterations in the topoisomerase IIα gene, messenger RNA, and subcellular protein distribution as well as reduced expression of the DNA topoisomerase IIβ enzyme in a mitoxantrone-resistant HL-60 human leukemia cell line. *Cancer Res.*, **55,** 1707–1716.

HOWARD MT, NEECE SH, MATSON SW AND KREUZER KN. (1994). Disruption of a topoisomerase-DNA cleavage complex by a DNA helicase. *Proc. Natl Acad. Sci. USA*, **91,** 12031–12035.

KALLIONIEMI OP, KALLIONIEMI A, KURISU W, THOR A, CHEN LC, SMITH HS, WALDMAN FM, PINKEL D AND GRAY JW. (1992). ErbB2 amplification in breast cancer analyzed by fluorescence in situ hybridization. *Proc. Natl Acad. Sci. USA*, **89,** 5321–5325.

KIMURA K, SAIJO M, UI M AND ENOMOTO T. (1994). Growth state- and cell cycle-dependent fluctuation in the expression of two forms of DNA topoisomerase II and possible specific modification of the higher molecular weight form in the M phase. *J. Biol. Chem.*, **269,** 1173–1176.

LEHRACH H. (1990). Genetic and physical mapping. In *Genome Analysis*. Vol. 1, Davies KE and Tilghman SM. (eds) pp. 39–81. Cold Spring Harbor Laboratory Press: New York.

LING V. (1992). P-glycoprotein and resistance against anticancer drugs. *Cancer*, **69,** 2603–2609.

LIU LF. (1989). DNA topoisomerase poisons as antitumor drugs. *Annu. Rev. Biochem.*, **58,** 351–375.

MA L, KRISHNAMACHARY N AND CENTER MS. (1995). Phosphorylation of the multidrug resistance protein gene encoded protein P190. *Biochemistry*, **34,** 3338–3343.

MCPHERSON JP, BROWN GA, DEUCHARS KL AND GOLDENBERG GJ. (1995). Increased sensitivity to adriamycin in drug-resistance P388 murine leukemia cells transfected with human Topoisomerase II$\alpha$. *Proc. Am. Assoc. Cancer Res.*, **36,** 2641.

MEIJER C, MULDER NH, TIMMER-BOSSCHA H, ZIJLSTRA JG AND DE VRIES EGE. (1987). Role of free radicals in an adriamycin-resistant human small cell lung cancer cell line. *Cancer Res.*, **47,** 4613–4617.

MÜLLER M, MEIJER C, ZAMAN GJR, BORST P, SCHEPER RJ, MULDER NH, DE VRIES EGE AND JANSSEN PLM. (1994). Overexpression of the gene encoding the multidrug resistant-associated protein results in increased ATP-dependent glutathione S-conjugate transport. *Proc. Natl Acad. Sci. USA*, **91,** 13033–13037.

MURPHY DS, MCHARDY P, COUTS J, MALLON EA, GEORGE WD, KAYE SB, BROWN R AND KEITH WN. (1995). Interphase cytogenetic analysis of erbB2 and topo$II\alpha$ co-amplification in invasive breast cancer and polysomy of chromosome 17 in ductal carcinoma in situ. *Int. J. Cancer*, **64,** 18–26.

NITISS JL, LIU YX, HARBURY P, JANNATIPOUR M, WASSERMAN R AND WANG JC. (1992). Amsacrine and etoposide hypersensitivity of yeast overexpressing DNA topoisomerase II. *Cancer Res.*, **52,** 4467–4472.

POMMIER Y, LETEURTRE F, FESEN MR, FUJIMORI A, BERTRAND R, SOLARY E, KOHLHAGEN G AND KOHN KW. (1994). Cellular determinants of sensitivity and resistance to DNA topoisomerase inhibitors. *Cancer Invest.*, **12,** 530–542.

TAN KB, DORMAN TE, FALLS KM, CHUNG TDY, MIRABELLI CK, CROOKE ST AND MAO JI. (1992). Topoisomerase-II$\alpha$ and topoisomerase-II$\beta$ genes - characterization and mapping to human chromosome-17 and chromosome-3, respectively. *Cancer Res.*, **52,** 231–234.

TIMMER-BOSSCHA H, HOSPERS GAP, MEIJER C, MULDER NH, MUSKIET FAJ, MARTINI IA, UGES DRA AND DE VRIES EGE. (1989). Influence of docosahexanoic acid on cisplatin resistance in a human small cell lung carcinoma cell line. *J. Natl Cancer Inst.*, **81,** 1069–1075.

VAN DER GRAAF WTA, DE VRIES EGE, TIMMER-BOSSCHA H, MEERSMA GJ, MESANDER G, VELLENGA E AND MULDER NH. (1994). Effects of amiodarone, cyclosporin A, and PSC 833 on the cytotoxicity of mitoxantrone, doxorubicin, and vincristine in non-P-glycoprotein human small cell lung cancer cell lines. *Cancer Res.*, **54,** 5368–5373.

VAN DER ZEE AGJ, WITHOFF S, KRANS M AND DE VRIES EGE. (1995). DNA topoisomerase I, II$\alpha$ and II$\beta$ protein and RNA expression levels in human ovarian carcinoma. *Proc. Am. Assoc. Cancer Res.*, **36,** 2671.

VERSANTVOORT CHM, WITHOFF S, BROXTERMAN HJ, KUIPER CM, SCHEPER RJ, MULDER NH AND DE VRIES EGE. (1995). Resistance associated factors in human small cell lung carcinoma $GLC_4$ sublines with increasing adriamycin resistance. *Int. J. Cancer*, **61,** 375–380.

WITHOFF S, SMIT EF, MEERSM GJ, VAN DEN BERG A, TIMMER-BOSSCHA H, KOK K, POSTMUS PE, MULDER NH, DE VRIES EGE AND BUYS CHCM. (1994). Quantitation of DNA topoisomerase II$\alpha$ messenger ribonucleic acid levels in a small cell lung cancer cell line and two drug resistant sublines using a Polymerase Chain Reaction-aided transcript titration assay. *Lab. Invest.*, **71,** 61–66.

WITHOFF S, KEITH WN, KNOL AJ, COUTTS JC, HOARE SF, MULDER NH AND DE VRIES EGE. (1996). Selection of subpopulation with fewer DNA topoisomerase II$\alpha$ gene copies in a doxorubicin-resistant cell line panel. *Br. J. Cancer*, **74,** 502–507.

WOESSNER RD, CHUNG TDY, HOFMANN GA, MATTERN MR, MIRABELLI CK, DRAKE FH AND JOHNSON RK. (1990). Differences between normal and ras-transformed NIH-3T3 cells in expression of the 170 kD and 180 kD forms of topoisomerase II. *Cancer Res.*, **50,** 2901–2908.

WOESSNER RD, MATTERN MR, MIRABELLI CK, JOHNSON RK AND DRAKE FH. (1991). Proliferation- and cell-dependent differences in expression of the 170 kilodalton and 180 kilodalton forms of topoisomerase II in NIH-3T3 cells. *Cell Growth Diff.*, **2,** 209–214.

YAMAZAKI K, ISOBE H, OGURA S AND KAWAKAMI Y. (1995). Topoisomerase II$\alpha$ content and topoisomerase II catalytic activity cannot explain drug sensitivities to topoisomerase inhibitors in lung cancer cell lines. *Proc. Am. Assoc. Cancer Res.*, **36,** 2663.

ZAMAN GJR, FLENS MJ, VAN LEUSDEN MR, DE HAAS M, MULDER HS, LANKELMA J, PINEDO HM, SCHEPER RJ, BAAS F, BROXTERMAN HJ AND BORST P. (1994). The human multidrug resistance-associated protein MRP is a plasma membrane drug-efflux pump. *Proc. Natl Acad. Sci. USA*, **91,** 8822–8826.

ZIJLSTRA JG, DE VRIES EGE AND MULDER NH. (1987). Multifactorial drug resistance in an adriamycin-resistant human small cell lung carcinoma cell line. *Cancer Res.*, **47,** 1780–1784.

ZINI N, SANTI S, OGNIBENE A, BAVELLONI A, NERI L-M, VALMORI A, MARIANI E, NEGRI C, ASTALDI-RICOTTI GCB AND MARALDI NM. (1994). Discrete localization of different DNA topoisomerases in HeLa and K562 cell nuclei and subnuclear fractions. *Exp. Cell Res.*, **210,** 336–348.